# Melorheostosis - a rare and progressive disease

**DOI:** 10.11604/pamj.2023.44.69.38954

**Published:** 2023-02-06

**Authors:** Suyash Yashwant Ambatkar, Megha Dipak Rudey

**Affiliations:** 1 Department of Orthopedics, Datta Meghe Institute of Medical Sciences (DU), Sawangi, Wardha, Maharashtra, India,; 2 Department of Kaumarbhritya, Mahatma Gandhi Ayurveda College, Hospital and Research Centre, Salod (H), Datta Meghe Institute of Medical Sciences (DU), Sawangi, Wardha, India

**Keywords:** Melorheostosis, rare, candle wax disease

## Image in medicine

Melorheostosis is a rare and progressive disease characterized by the thickening or widening of the cortical bone. It affects bone as well as soft tissue growth and development. The disorder is benign but often results in severe functional limitation. The incidence of the disease is 1: 10,00,000. Both males and females are affected, only 400 cases have been reported until now. A 1-year-old male child was brought to the orthopedics outpatient department (OPD) with complaints of pain and contractures in his right forearm which were increasing with the age of the patient, with no history of medical illness or trauma. On examination, there were soft tissue contractures on the right hand and abnormal cortical thickening of the radius and ulna bones was noted. X-ray of the right forearm with elbow anterior-posterior (AP) and lateral was done which showed sclerosis of the bone along with a characteristic candle wax appearance.

**Figure 1:**
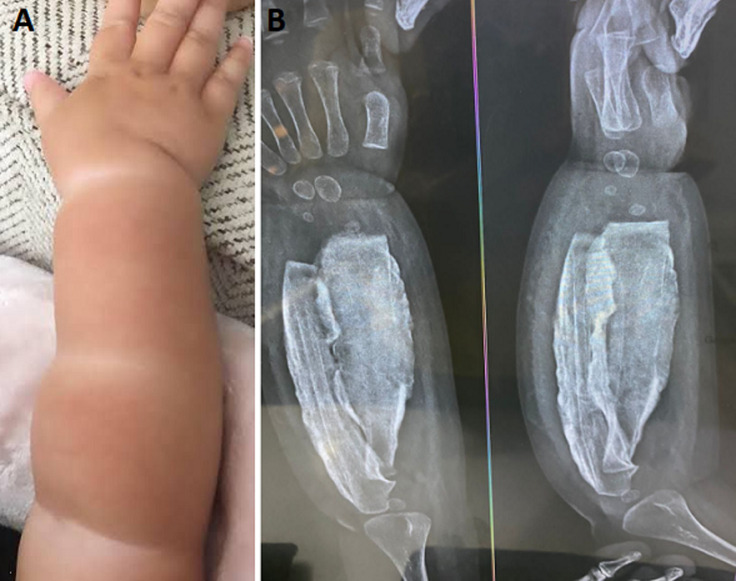
A) multiple soft tissue contractures in the child’s right hand; B) sclerosis of the bone with characteristic dribbling candle wax appearance over the radius and ulna

